# An *atonal* homolog, *lin-32,* regulates hypodermal morphogenesis in *Caenorhabditis elegans*

**DOI:** 10.17912/micropub.biology.000754

**Published:** 2023-02-14

**Authors:** Sayaka Hori, Shohei Mitani

**Affiliations:** 1 Tokyo Women's Medical University, Tokyo, Tokyo, Japan; 2 Physiology, Tokyo Women's Medical University, Tokyo, Tokyo, Japan

## Abstract

The transcription factor *atonal* contributes to patterning and cell fate determination in specialized epithelial cells in various animals, but its function in hypodermis is unknown. Here, we analyzed the *atonal* homolog *lin-32* in *C. elegans* to clarify whether *atonal* acts in hypodermal development. The *lin-32* null mutants exhibited bulges and cavities in their head, which were prevented by LIN-32 expression. Fluorescent protein was expressed in hypodermis cells at the embryonic stage by the *lin-32* promoter. These results certify that *atonal* plays an essential role in the development of a broader range of tissues as hypodermis than initially thought.

**Figure 1. Expression and function of lin-32 in hypodermal cells.**
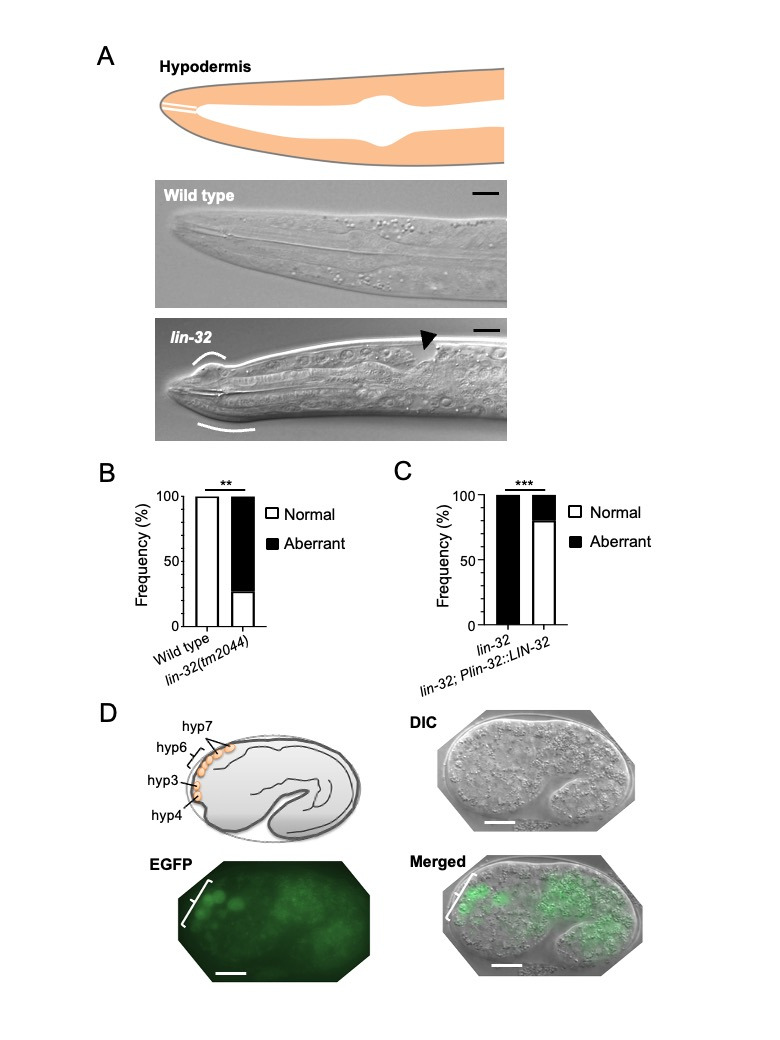
(A) The *lin-32* null mutants showed various morphologies of head hypodermis. Localization of the hypodermis on left lateral view (orange-colored area). The wild-type animal had a smooth head shape in a differential interference contrast (DIC) image. The *lin-32* null mutants exhibited abnormal head shape. The nose tip was curved (white lines) and a cavity was observed (arrowhead). (DIC images). Scale bars = 100 µm. (B, C) The *lin-32* gene is necessary for head morphogenesis. (B) All wild-type animals (100%) presented standard heads (n = 6), but 81.2% of the *lin-32* null mutants exhibited a change in head morphology (“Aberrant,” n = 11). **p = 0.0090 < 0.01 (Fisher's exact test). (C) Rescue experiment. Head morphological changes in *lin-32* null mutants (0% normal, n = 20) were rescued by LIN-32 expression from its own promoter (80.0% normal, n = 25). ***p < 0.0001 (Fisher's exact test). (D) Schematic diagram of hypodermal cell localization (hyp3, hyp4, hyp6, and hyp7) on the dorsal anterior side of the head primordium of a 1.5-fold stage embryo (Sulston et al. 1983). DIC image of a 1.5-fold stage embryo. Fluorescence image of EGFP expressed by the *lin-32* promoter. EGFP expression was observed in dorsal hypodermal cells centered around hyp6. Scale bar (white bracket) = 10 µ m.

## Description

Here, we provide new insights into the basic helix-loop-helix transcription factor *atonal* in hypodermis development. Historically, *atonal* had been identified as a proneural gene, contributing to neural fate determination in various animals. For example, in *Caenorhabditis elegans* ( *C. elegans* ) *,* the *atonal* homolog *lin-32* initiates the neurogenesis touch neurons (Chalfie and Sulston 1981; Mitani et al. 1993). We also have previously reported that *lin-32* is essential for optimizing avoidance behaviors via AIB interneuron development (Hori et al. 2018). The *atonal* gene and its homolog also contribute to patterning and cell fate determination in specialized epithelial cells like those in the retina (Robertson et al. 2012), intestine (VanDussen and Samuelson 2010), and the inner ear sensory epithelia (Bermingham et al. 1999). However, its function in the hypodermis remains unclear.

The epithelial system of *C. elegans* is constituted by the hypodermis and specialized epithelial cells. In this study, to determine the role of *atonal* in hypodermis development, we examined the morphology of the head hypodermis in *lin-32* null mutants. *lin-32* null mutants exhibited morphological changes in the head hypodermis, presenting external bulges and an internal cavity (Fig. 1A). The phenotype was rescued by the expression of LIN-32 driven by its own promoter (Fig. 1B,C). DNA microarray analysis revealed that *lin-32* is expressed in a restricted cell lineage within a narrow time window during embryogenesis (Murray et al. 2012). In the hypodermis, *lin-32* is predicted to be expressed in hyp4 and hyp6, which are derived from the ABarpapa lineage (Murray et al. 2012). To assess *lin-32* -expression in the peripheral area in embryos, we used a *lin-32* promoter-driven EGFP and have observed fluorescence in hyp6 and additional cells that have not yet been identified. These results provide insight into the function of *lin-32* in the differentiation of the hypodermis. However, we could not identify the causal cell lineage that induced morphological changes in the head, because the number and location of the hypodermal nuclei were substantially altered in the *lin-32* null mutants. Considering the principal role of hyp6 in the morphology of the anterior hypodermis (Cinar and Chisholm 2004), we speculate that *lin-32* also affects hyp6 development or localization. These findings reveal a novel role of *atonal* , which is well known as a proneural gene, in a broader range of tissues than initially thought.

## Methods

Nematodes and maintenance

We cultured *C. elegans* strains using modified standard techniques as described previously (Brenner 1974; Hori et al. 2018). The *lin-32* ( *tm2044* ) mutants were backcrossed with the wild-type animal N2, five times. The information on the strains used is summarized in Table 1.

Microscopy

*C. elegans* adults were immobilized with M9 buffer containing sodium azide 50 mM on a 5% agarose pad containing 10 mM sodium azide (Wako, 195-11092) (Fig. 1 A,B,C). Eggs were isolated from gravid adults by treating them with a bleaching solution (Fig. 1D). The bleaching solution contained 0.2 mL 4N NaOH (Wako, 194-18865), 0.3 mL 6% bleach (Kao, o17246), and 0.5 mL M9 buffer. Differential interference contrast (DIC) and fluorescence images were obtained using a BX51 microscope equipped with a DP30BW charge-coupled device (CCD) camera (Olympus Optical).

Plasmid construction

In the own promoter rescue experiment (Fig. 1C), pFX_P *lin-32* ::LIN-32 was used (Hori et al. 2018). To analyze *lin-32* expression (Fig. 1D), pFX_P *lin-32* (4.2K)::EGFP was constructed by cloning the *lin-32* promoter (from 4.2 kbp of the genomic sequence upstream of the mature *lin-32* sequence), which was amplified using PCR, with the N2 genomic DNA as the template. Cloning primers are as follows; ggttccgcgtggatccCTGAAAATTAGAAACTAAATGAG, GCTCACCATgcggccgcCCATGGTTGGTCTGACTGAAAACGAC. The pFX_EGFP plasmid and the PCR fragment of the *lin-32* promoter were cut using two restriction enzymes, BamHI (Takara, 1010A) and NotI (Takara, 1166A), and then ligated using ligase (Takara, 6021).

Transgenic lines and strains

To generate *lin-32(tm2044);* *tmEx3143* transgenic animals (Fig. 1C), pFX_P *lin-32* ::LIN-32 (20 ng/μl), and pBluescript KS(+)T1(100 ng/μl) were co-injected, along with pFX_ *Pnmr-1::* mCherry (80 ng/μl), into FX15206 animals (Hori et al. 2018). To generate *tmEx3090* transgenic animals (Fig. 1D) *,* pFx_P *lin-32* (4.2 kb)::EGFP (150 ng/μl) was injected along with pFX_P *nmr-1* ::mCherry (50 ng/μl), which acts as an injection marker, into N2 animals.

Quantification and statistical analysis

Statistical analyseswere performed using the GraphPad Prism 6 software (GraphPad software). Pairwise comparisons of frequencies within two groups wereperformed using Fisher’s exact test (Fig. 1B,C).In both analyses,theDIC images were randomized and their normalcy was ascertained in a blinded manner.Figures 1B and 1C are the experimental results of sampling on different days. There is a variable penetrance of incidence and severity of the morphological defect in the *lin-32* mutants(Fig. 1B,C). Therefore, we speculate that the penetrance is responsible for the difference in the two results(Fig. 1B,C).The statistical information and the total number of animals analyzed per experiment are indicated in Figure legend.

## Reagents

**Table:** 

Strain name	allele name	Genotype	Available from	Outcross	Reference
N2	-	*Caenorhabditis elegans* wild type (ancestral)	CGC	-	
FX15206	*tm2044*	lin-32(tm2044)	Mitani Lab	x 5	Hori S, et al., 2018.
FX15218	*tm2044 ; tmEx3143 *	lin-32(tm2044); tmEx3142[Plin-32::LIN-32 + Pnmr-1::mCherry + pBluescripts KS+T1]	Mitani Lab	x 5	Hori S, et al., 2018.
FX15181	*tmEx3090*	tmEx3090[Plin-32(4.2 kbp)::EGFP + Pnmr-1::mCherry]	Mitani Lab	-	This paper
					
Plasmid	Genotype	Description	Available from	Reference	
pFx_ *Plin-32::* LIN-32	*Plin-32::LIN-32*	5.1 kbp DNA fragment contained 4.2 kbp of *lin-32* promoter and cording region.	Mitani Lab	Hori S, et al., 2018.	
pFx_ *Plin-32(4.2K)::* EGFP	*Plin-32::EGFP*	4.2 kbp DNA fragment (lin-32 promoter) was joined to EGFP.	Mitani Lab	This paper	
pFx_ *Pnmr-1::* mCherry	*Pnmr-1::mCherry*	5.0 kbp DNA fragment (nmr-1 promoter) was joined to mCherry.	Mitani Lab	Hori S, et al., 2018.	
